# A Scoring Model and Protocol to Adapt Universal Screening for Lynch Syndrome to Identify Germline Pathogenic Variants by Next Generation Sequencing from Colorectal Cancer Patients and Cascade Screening

**DOI:** 10.3390/cancers14122901

**Published:** 2022-06-12

**Authors:** Ramadhani Chambuso, Barbara Robertson, Raj Ramesar

**Affiliations:** 1 Colorectal Cancer Research Group, Division of Human Genetics, Department of Pathology, Faculty of Health Sciences, University of Cape Town, Cape Town 7701, South Africa; raj.ramesar@uct.ac.za; 2 MRC Unit for Genomic and Precision Medicine, Division of Human Genetics, Department of Pathology, Institute of Infectious Disease and Molecular Medicine, Faculty of Health Sciences, University of Cape Town, Cape Town 7701, South Africa; 3 Division of Radiation Oncology, Department of Radiation Medicine, Groote Schuur Hospital, University of Cape Town, Cape Town 7945, South Africa; barbara.robertson@uct.ac.za

**Keywords:** Lynch syndrome, colorectal cancer, Lynch syndrome scoring model, germline pathogenic variants, next generation sequencing, cascade screening, modified ascertainment and follow up program

## Abstract

**Simple Summary:**

Lynch syndrome (LS) is an autosomal-dominantly inherited form of cancer predisposition dominated by colorectal cancer (CRC). LS is caused by germline pathogenic variants (PV) occurring in known mismatch repair genes. For effective cascade screening, it is critical to identify PV for LS predisposition. When limited resources are available, next generation sequencing (NGS) of an entire cohort of colorectal cancer (CRC) patients, even those under 50 or 60 years of age, places a huge burden on the system. Here, we present an innovative LS ascertainment and follow-up program that includes LS molecular analysis, PV screening with NGS technology, and cascade screening. The goal is to improve LS ascertainment in light of the growing burden of early-onset CRC, particularly in low- and middle-income countries.

**Abstract:**

Identification of germline pathogenic variants (PV) predisposing to Lynch syndrome (LS) is an important step for effective use of cascade screening of extended at-risk lineages, leading to reduced morbidity and mortality due to colorectal cancer (CRC). As a general rule, however, next generation sequencing (NGS, either of gene panels or whole exomes) is relatively expensive and unaffordable for general clinical use. In resource-poor settings, performing NGS testing on an entire cohort of CRC patients, even if limited to those under 50 or 60 years of age, still places an enormous burden on limited resources. Although family history can be a good indicator for LS testing, identifying at-risk family members and offering cascade screening may not benefit many patients/probands without an obvious family history. This article presents a novel program called Modified Ascertainment and follow-up Program (MAP) with a scoring model for LS ascertainment and molecular screening by NGS with diagnosis confirmation of PV and cascade screening. The goal is to improve LS ascertainment in light of the growing burden of early-onset CRC, particularly in low- and middle-income countries. Through MAP, judiciously applied molecular genetics will improve identification of PV predisposing to LS and cascade screening.

## 1. Introduction

Germline pathogenic variants (PV) occurring in the known mismatch repair genes, namely *MSH2*, *MLH1*, *MSH6*, *PMS2*, or deletions of the *EPCAM* gene, result in Lynch syndrome (LS). LS is an autosomal-dominantly inherited form of cancer predisposition, dominated by colorectal cancer (CRC) which accounts for up to 80% of the primary tumor site [1,2]. About 5% to 10% of all CRC cases are caused by high penetrance familial cancer syndromes, including the LS [3]. Approximately 2% to 3% of all CRC cases are caused by LS, the most common inherited cancer syndrome [4].

The majority of LS-variant heterozygotes develop colorectal, endometrial, ovarian, breast (in females) and prostate cancers [5]. Moreover, a significant proportion of LS-variant heterozygotes also develop cancers at other sites than that observed in the general population. Germline variants in genes associated with cancer susceptibility increase the risk of developing CRC before age 60 years [6]. The prevalence of PV generally ranges from 20–30% in young CRC patients under 60 years of age [7,8]. An evidence-based precision medicine approach called ‘Universal Screening for LS’, which includes molecular genetic testing, has been recommended internationally to identify LS variant heterozygotes [9,10,11]. When LS is routinely identified and ‘universal screening’ is applied to extensive genetic lineages, where variant heterozygotes are ascertained pre-symptomatically and appropriately managed, significant improvement in CRC disease outcomes can be achieved amongst those at highest risk [8,12]. Furthermore, we have shown in our previous work that chemoprevention using aspirin and the intensive screening backup plan do help to decrease CRC incidence in LS-variant heterozygotes [13]. Technically this is achieved through clinical surveillance of relatives of asymptomatic LS-variant heterozygotes. Detection of very early lesions and their removal greatly reduces morbidity and mortality [14].

Cascade screening of asymptomatic high-risk relatives (through genetic counselling and DNA sequencing [usually by next generation sequencing, (NGS)] to confirm LS diagnosis present a variety of technical, logistical, psychosocial, and economic challenges, including:

- Who should and should not undergo NGS for LS?
- How can precision medicine be applied to increase NGS testing for LS in limited resources?
- How can NGS testing be made more affordable and cost-effective for LS?
- What contribution can precision medicine and molecular genetics make to improve cascade screening for LS?

In an ideal clinical setting, PV responsible for LS (in a given family) should be identified in the proband (i.e. primary family member affected with a likely LS-attributable cancer). In such a setting, the application of cascade screening with the known PV will have near 100% detection-rate in at-risk individuals [15]. The application of genetic testing by NGS, is most effectively carried out under the circumstance of a high index of suspicion for LS; this takes into account a range of previously used guidelines including Bethesda [16] and NICE [17], which includes a relatively young age at diagnosis (<50 years) and a family history. Genetic counselling is recommended before genetic testing is performed [15,18].

For example, in hereditary breast and ovarian cancer (*BRCA* variants), genetic testing involving the unaffected close relative is recommended when the statistical screening model indicates a risk of 10% or more for PV identification [19]. Testing for PV for LS based on such a priori risk has, to date, found little support. A combination of the limited number of statistical or predictive models for LS and the lower tendency toward patient-centered advocacy may explain why this is the case. If we identify a significant proportion of CRC patients with LS and then establish cascade screening and detect neoplasms early, before symptoms appear, mortality and morbidity due to CRC among family members is known to be reduced [20,21,22,23,24,25,26,27].

To address the above concerns and limitations especially in resource-constrained settings, we present an innovative program called the Modified Ascertainment and follow-up Program (MAP). MAP model assigns a score for ascertainment of LS from the relatively large burden of CRC patients, and the score indicates if one should proceed with molecular confirmation of PV using NGS and subsequent cascade screening.

## 2. Lynch Syndrome Testing Decision

A combination of considerations is required to answer the question of who is eligible for PV testing using NGS. These include whether the disease runs in the family, the likelihood that the eligible patient is a variant heterozygote, and the degree of sensitivity of the variant test [28,29].

Microsatellite instability (MSI) or immunohistochemistry (IHC) can be used for LS pre-screening [30]. Clinical presentation alone does not lead to a diagnosis. Even when clinical presentation and family history are most convincing, the yield from variant testing is often less than 50% [28]. If all strict clinical criteria, i.e., Amsterdam I and II criteria (AC), are met and a tumor has high MSI or loss of MMR protein expression, the probability of detecting a germline mutation increases significantly to approximately 70% to 80% [31]. NGS testing is expensive and is often not reimbursed by health insurers, therefore the development of a relatively low-cost schema to identify the most likely LS-variant heterozygotes would assist with the decision to proceed with NGS.

**Figure 1:**
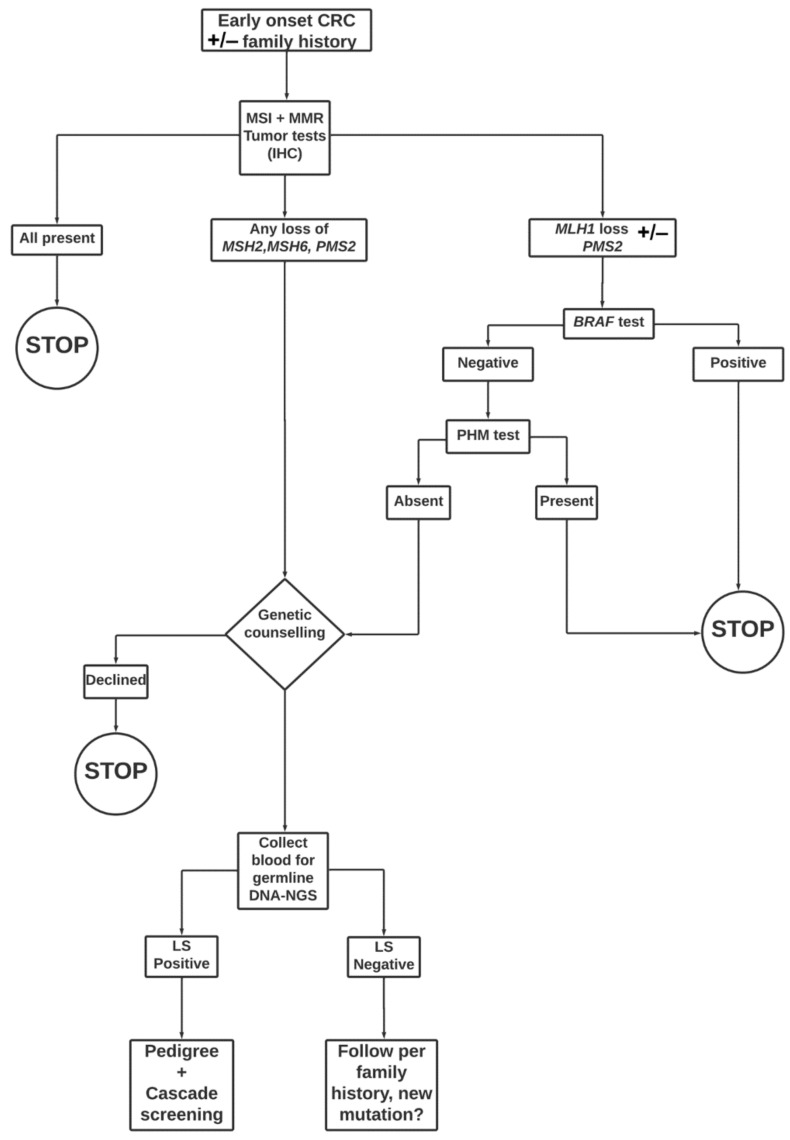
Workflow illustrating the process of genetic diagnosis of LS. A patient presents with early-onset CRC (age equal to/below 60 years). This presentation alone or in combination with other findings (family history, clinical and pathologic features of the tumor) raises suspicion that LS may be present. The tumor is examined for evidence of MSI (Bethesda marker), or lack of MMR protein expression (using IHC) [34,35]. If these findings are conclusive, they are supplemented by germline DNA mutation testing using NGS.

A genetic diagnosis of LS is usually made in three steps. The first step is for the patient to present with an early-onset CRC. When this occurs, whether alone or in combination with other observations (namely: family history, clinical and pathologic features of the tumor), LS is suspected. Second, tumors are screened for signs of MSI or absence of MMR proteins using IHC. If these findings are conclusive, they are supplemented in a third step by NGS testing for pathological variants [17,32,33] (Figure 1). Recently, there have been several attempts to shed light on which clinical predictors are most likely to be useful. Studies have also compared the yield of MSI and IHC as alternative or complementary approaches in different clinical settings [32].

A family history of cancer and the patient’s age at diagnosis of CRC are recommended by the AC. The revised Bethesda Guidelines (BG) were designed to determine in what tumor types MSI testing should be performed. This generally encompassed the full spectrum of LS tumors, as well as a range of histopathologic features. Approximately 90% of individuals found to have MMR germline variant, had MSI detected between clinical suspicion and variants testing [36]. The negative predictive value is particularly important since pathologic MMR variants are rarely found in tumors that do not have MSI. Testing with MSI can improve the decision-making process and increase the chance of detecting mutations by up to 70%. As a result of the introduction of IHC for the DNA MMR proteins, public hospitals in South Africa can now test for loss of expression of the culprit proteins and perform targeted genetic testing. MSI testing and IHC have been shown to have excellent correlation. IHC should be performed first and in a sequential manner before MSI testing. There is a possibility of cost savings if IHC is informative since it is specific for the MMR gene whose expression has been lost (as a result of a PV). MSI can be performed if the IHC is not informative.

Tumor screening with MSI and/or IHC is the preferred method recommended by the National Comprehensive Cancer Network (NCCN). A caveat needs to be highlighted in both cases of MSI and/or IHC, use. While most LS tumors have MSI alterations, this is also true in approximately 12% to 15% of sporadic CRC. In sporadic MSI, it is almost always the case that the *BRAF* V600E mutation and hypermethylation of the *MLH1* promoter are present, whereas these features are rarely, if ever, present in LS. *BRAF* and *MLH1* promoter hypermethylation can be detected using commonly available tests. It is not possible to diagnose LS based on MSI or IHC alone, since IHC can detect mutations in a few MSI-negative tumors, but other MSI-positive tumors are missed.

To make a meaningful molecular genetic diagnosis for LS, one should routinely combine the best clinical practice including the patient’s family history, pathologic, and molecular genetic findings. At present this remains one of the biggest challenges to ascertaining individuals with familial CRC which may be eligible for LS genetic testing for PV by NGS.

## 3. Local Challenges and LS Screening in Resource-Limited Settings

Since the early 1990s, a multidisciplinary team of surgeons, oncologists, pathologists, geneticists, and gastroenterologists at the University of Cape Town (and affiliated hospitals) in South Africa has been caring for families with LS [26,37]. In the Western and Northern Cape provinces of South Africa, identifying individuals with hereditary cancers and managing them according to their empiric risk was effective even before genetic testing became available. Emerging from this work, we currently have records of 450 families with LS currently receiving care. The CRC outcomes of these families have improved significantly since they started with confirmed index cases of CRC that were genetically tested and shown to carry PV, and where the family was offered genetic counselling and cascade testing, and clinical surveillance. With our multidisciplinary LS management group at the University of Cape Town, we have shown that managing individuals with pre-symptomatic genetic testing and clinical surveillance is more cost-effective than treating disease when it is already present [38,39,40]. In South Africa, there is a commitment to evidence-based interventions in a localized application of universal screening for LS. Despite the program of managing LS families described in this section, the genetic referral system and NGS testing is not fully functional in Cape Town, and it is even less effective in other parts of South Africa [22,41,42]. Therefore, we need to improve our strategies to identify CRC patients with PV for LS and expand a reliable, efficient and cost-effective multidisciplinary CRC network to other centers in South Africa to reduce morbidity, mortality and healthcare costs [14,22,43]. In light of the above, it is necessary to explore the following questions:

- Why do a significant number of LS patients, mutation carriers and their siblings die from CRC?
- Can anything be done to improve the ascertainment of LS and prevent these deaths?
- Is it possible that among our LS patients, asymptomatic variant heterozygotes and their families who have benefited from genetic testing and predictive management, there were cases that were missed and/or not followed up?
- IWhat are the potential outcomes of LS patients, their quality of life, and the cancer risk status of their children and siblings?

## 4. The Modified Ascertainment Program (MAP) Scoring Model and Protocol for LS Screening

**Figure 2:**
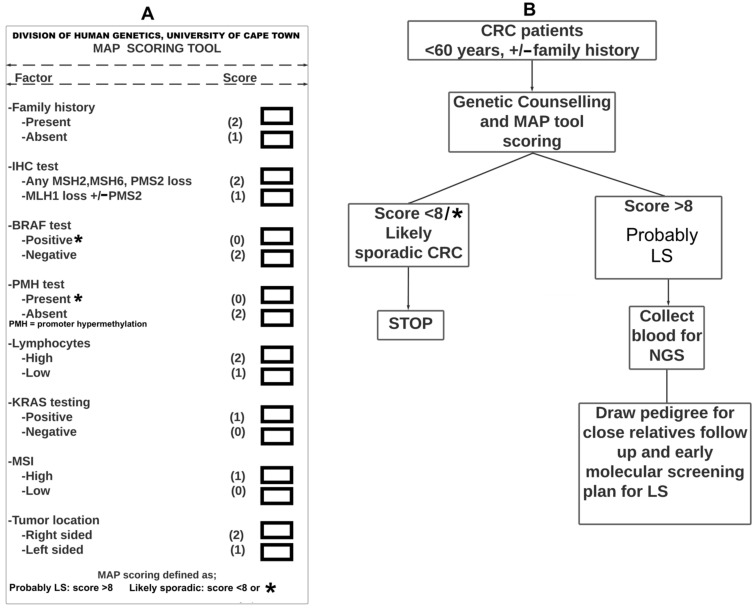
The proposed MAP for screening of CRC patients for PV for LS by NGS. (**A**) The MAP scoring tool/model assigns scores of 0, 1, or 2 to known factors used to diagnose LS. If the total score is below the cut-off point of 8, the patient likely has sporadic CRC, if the total score is above 8, the patient probably has LS, and finally, if *BRAF* is positive or *MLH1* promoter hypermethylation is present, the patient likely has sporadic CRC. (**B**) The MAP protocol for CRC patients under 60 years of age regardless of a family history. The process begins with genetic counselling for patient consent, followed by the MAP scoring model. If the total score is less than 8 or *BRAF* positive or *MLH1* promoter hypermethylation is present, LS testing should be terminated. If the total score is greater than 8, blood collection for NGS and drawing of pedigree, and cascade screening should follow. Amsterdam II criteria for LS should be followed for family history. There should be at least three relatives with any LS-associated cancer (CRC, cancer of the endometrium, small bowel, ureter, or renal pelvis). One should be a first-degree relative of the other two. At least two successive generations should be affected [31]. * shows BRAF test positive or PMH is present.

The MAP screens all CRC patients equal to or under 60 years old at diagnosis using a locally developed scoring tool/model and protocol, as illustrated in (Figure 2A,B, respectively). Also, it ascertains likely LS-variant heterozygotes from a cohort of CRC patients, and their subsequent targeted molecular diagnosis. Furthermore, it follows-up the pre-symptomatic family members with genetic counselling, genetic testing by NGS and clinical surveillance, which is innovative in our setting [17,33]. This cost-effective novel approach will increase identification of probands [44,45]. NIH has received a MAP grant application with reference number R01 GRANT13543177/2022 that has been submitted for funding consideration. This is in collaboration with three of the largest public hospitals in South Africa. These hospitals have agreed to adopt our MAP model for LS screening. The proposal will examine in detail the barriers and facilitators to implement MAP in these hospitals, which serve four of the country’s nine provinces.

## 5. Significance of the MAP Model/Tool for LS Screening

Compared to developed countries, Africa has fewer than 10 health professionals per 1000 population, which emphasizes that specialized resources for screening LS are unevenly distributed around the continent. It is against this backdrop, that innovative interventions are required. In this regard, using the MAP tool we propose to screen out all CRC patients under 60 years of age and genetically test only those with a MAP score of 8 or more using NGS. LS screening for all CRC patients under 50/60 years of age or for all CRC patients, as in some developed and first-world countries, both differ from the MAP approach [28,46]. When resources are limited, MAP, involving optimal use and scoring of demographics, clinical (including Amsterdam II criteria family history), and histopathological data is a cost-effective and time-saving solution for ascertainment of LS in amongst the affected CRC population. In addition to timely ascertainment of LS and its clinical management in patients already affected with CRC, our proposed model will have a significant impact on the disease outcomes of their at-risk close relatives. CRC late presentation, mortality and morbidity can be reduced by increasing the likelihood of early genetic screening, diagnosis and follow-up. As a result of the MAP approach, not only will screening behavior change, but the ability to implement health care reform related to genomic medicine will be strengthened. MAP offers the following advantages on screening for PV for LS:

- Triage tool for genetic screening for PV by NGS
- Combination of multidisciplinary teams involved in LS/CRC management
- High accuracy in the identification of probands with PV
- Saving of time and labor
- Acceleration of cascade screening process
- Early molecular-based screening for relatives of LS patients
- Voluntary LS ascertainment and cascade screening initiated by the healthcare provider
- Diagnosis of LS with high precision
- Early colonoscopy screening for relatives at risk who tested positive for PV
- Reduction in the workload for genetic screening
- Reduction of mis-diagnosis of LS cases
- Reduction of costs associated with NGS screening indiscriminately
- Genetic classification of all <60 years CRC into sporadic and hereditary
- The MAP scoring model can be applied retrospectively in a CRC database
- Translating scientific innovation into clinical practice.

MAP is currently being optimized at Groote Schuur Hospital and two other large public hospitals that together provide health care to CRC patients in four of South Africa’s nine socio-demographically diverse provinces. This approach is designed to be suitable for use in sub-Saharan Africa and has the potential to be expanded throughout South Africa.

## 6. Genetic Counselling and Cascade Screening Using MAP Approach

LS should be screened in all CRC patients at the time of diagnosis for the presence of tumor features characteristic of LS. Whether or not there is a family history of CRC, all patients with tumors suspected to be LS should be referred to a genetic counselling service. Those with a high index of suspicion should be recruited for genetic testing. After identification of PV in an index CRC patient, cascade screening should be initiated, including genetic counselling and testing of blood relatives. Pre-symptomatic (at-risk) individuals, should be genetically tested, and offered colonoscopic surveillance if found to be variant heterozygotes for LS. Optimizing this approach is critical to reach a large number of LS families [47].

As an example of personalized oncology suggested by MAP, LS-associated CRC can be prevented and treated with a collaborative multidisciplinary effort between patients, physicians, and healthcare authorities. Hereditary cancer prevention and genetic testing should be designed to improve communication among at-risk relatives about hereditary cancers, at least for common hereditary cancer syndromes such as LS. Personalized CRC screening for LS by MAP and prevention plans should include cascade screening of blood relatives of known LS cases. However, at-risk relatives do not get genetically tested, which is most likely due to family members not being aware of inherited cancer syndromes, and even less about genes. An estimated 5% to 20% of all cancers are caused by PV in genes that are passed from generation to generation. PV detected in an index case should result in relatives being tested for the same variants. Their healthcare providers will use this information to create targeted cancer screenings and prevention plans.

Prior to genetic testing, at-risk individuals should receive genetic counselling so that they understand both the advantages and disadvantages of genetic testing and can thus provide fully informed written consent. The patient must be able to decide whether testing is in his or her personal and emotional best interest. In addition, the patient needs to feel confident about confidentiality issues that may be related to perceptions of discrimination in insurance or employment. The patient’s emotional state should also be considered by the genetic counsellor in order to accept a positive or negative test result. In this task, it is most effective if patients are familiar with their own family history of CRC, and are prepared to be tested. The genetic counsellor may notify the patient’s family (usually in conjunction with the index patient) if they consent. A key aspect of optimizing hereditary CRC detection and treatment is identifying all relevant stakeholders (from community healthcare workers to health care professionals) and improving communication mechanisms and recognizing evidence-based best practices. Public engagement, presentations at professional meetings, education, and training are all part of this communication. Our MAP approach emphasizes the involvement of multidisciplinary teams including oncologists, surgeons, cancer geneticists, gastroenterologists or informed primary care physicians, as well as genetic counsellors and registered nurses, to be part of the team that notifies and assesses LS at-risk family members. This multipronged approach will help reach as many at-risk family members as possible and provide them with information about hereditary cancer syndromes, DNA testing, cancer surveillance, and risk management.

## 7. Conclusions and Onco-Economics Perspective

As a result of the unequal distribution of resources, screening for LS varies widely around the world. The MAP scoring model screens all CRC patients 60 years of age or younger. However, only those who score 8 or above are offered with PV for genetic testing using NGS. This is both cost-effective and time-saving and is particularly relevant for resource-limited settings. A close relative without symptoms of CRC will benefit from this approach by cascade screening. Subsequent relatively inexpensive cascade genetic testing for known PV and clinical surveillance of individuals who test positive for the variants may reduce CRC late presentation and the associated mortality and morbidity. The MAP approach has the potential to reduce cancer care costs due to LS and facilitate future CRC prevention. Due to the high cost of commercial NGS, the MAP scoring model has a significant economic impact. Precision medicine, molecular genetic testing, and cascade screening can make a significant contribution to LS diagnosis, targeted screening, and treatment of families at risk for CRC.

## Data Availability

Data sharing not applicable. No new data were created or analyzed in this study. Data sharing is not applicable to this article.
